# SGLT2 Inhibitors, GLP-1 Agonists, and DPP-4 Inhibitors in Diabetes and Microvascular Complications: A Review

**DOI:** 10.1155/2020/1762164

**Published:** 2020-02-29

**Authors:** Christopher El Mouhayyar, Ruba Riachy, Abir Bou Khalil, Asaad Eid, Sami Azar

**Affiliations:** ^1^Department of Anatomy, Cell Biology and Physiology, American University of Beirut, Beirut, Lebanon; ^2^Diabetes Program, American University of Beirut-Medical Center, Beirut, Lebanon; ^3^Department of Internal Medicine, Division of Endocrinology and Diabetes, American University of Beirut-Medical Center, Beirut, Lebanon

## Abstract

The prevalence of diabetes and its associated complications is increasing throughout the decades. Promising diabetes medications were introduced to the market including GLP-1 agonists, DPP-4 inhibitors, and SGLT2 inhibitors aiming to target these complications. The literature lacks sufficient data regarding these new medications and their influence on nephropathy, retinopathy, and neuropathy. This review expands on the major results of effects of the 3 drug classes on microvascular complications. In our review, both SGLT2 inhibitors and GLP-1 agonists appear to have promising nephroprotective outcomes at this stage, with less promising outcomes seen with DPP-4 inhibitors. Moreover, the retinoprotective outcomes of both SGLT2 inhibitors and DPP-4 inhibitors were only tested on mice, while those of GLP-1 agonists were assessed in few trials. In addition, the results of both GLP-1 agonists and DPP-4 inhibitors showed discrepancies in these studies. On the contrary, conclusions regarding the effect of these medications on neuroprotective outcomes cannot be drawn at the time due to the lack of clinical trials targeting these complications. Hence, a clearer picture of the microvascular outcomes will manifest over time with the release of multiple upcoming clinical trials.

## 1. Introduction

**Figure 1:**
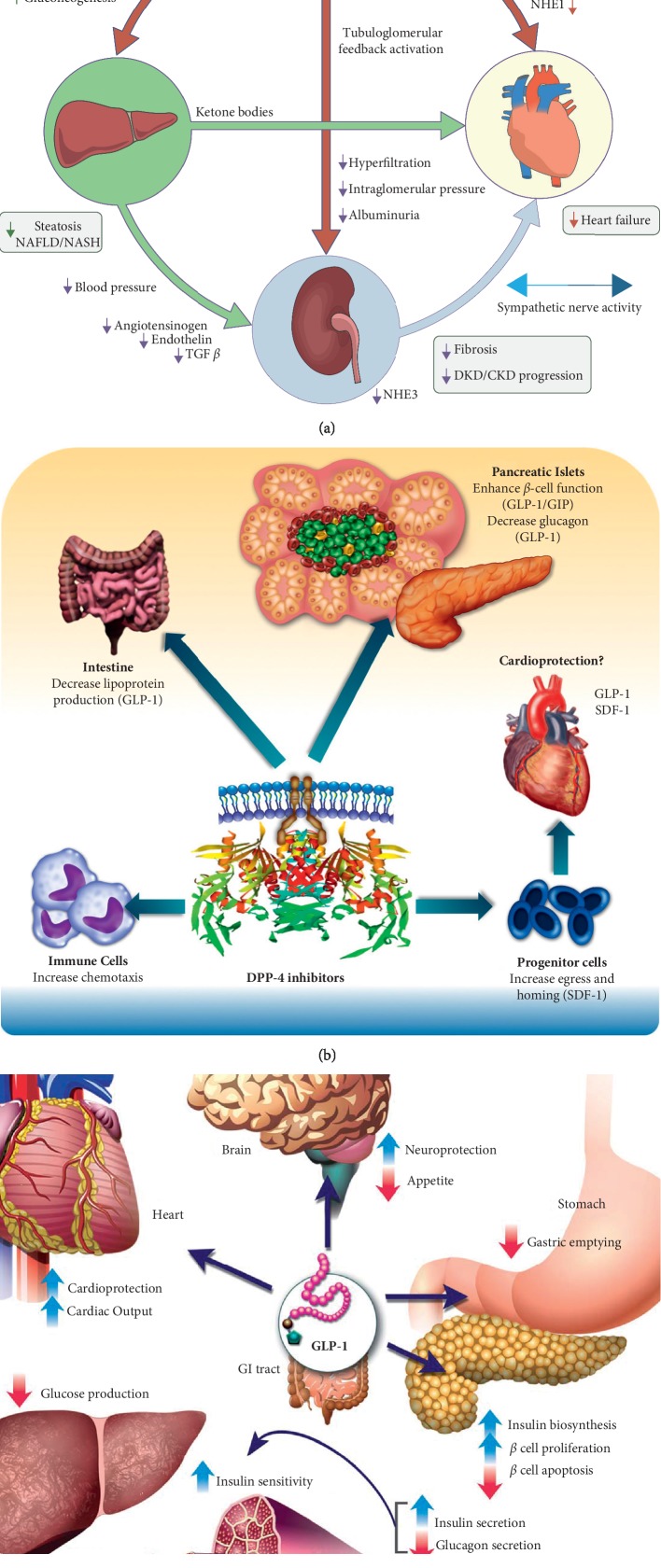
Mechanism of action of diabetes medications on multiple organ systems: (a) SGLT2 inhibitors [1]; (b) DPP-4 inhibitors [2]; (c) GLP-1 agonists [3].

During the 19^th^ century, the discovery of insulin constituted the landmark of the era in terms of glucose control. Although insulin was capable of controlling glucose levels, it lacked the protective effects that scientists strived to achieve. Moreover, patients on insulin are at risk of hypoglycemia and lipodystrophy which hinders their compliance. This has enticed the search for easier and safer drugs with an additional protective effect other than glucose control. Multiple drugs were introduced to the market including glucagon-like peptide-1 (GLP-1) agonists since 2005, dipeptidyl peptidase 4 (DPP-4) inhibitors since 2006, and sodium glucose cotransporter 2 (SGLT2) inhibitors since 2013. The effect of these medications on multiple organ systems is summarized in Figure 1.

To date, the current clinical trials show special interest in the effect of antidiabetic medications on macrovascular complications and mortality. The literature lacks sufficient data regarding new antidiabetic medications and their influence on nephropathy, retinopathy, and neuropathy. This paper is among a few to tackle the effect of 3 classes of antidiabetic medications on microvascular complications. In our paper, we included the main published data in MEDLINE and PubMed journals about this topic. We included results from both human and animal studies.

## 2. Nephropathy

### 2.1. SGLT2 Inhibitors and Nephropathy

Evidence has shown that SGLT2 inhibitors in addition to lowering glucose levels exert a protective effect at the microvascular and macrovascular levels. In particular, the EMPA-REG OUTCOME trial (Empagliflozin, Cardiovascular Outcomes, and Mortality in Type 2 Diabetes) has shown that empagliflozin reduced the risk of incidence or worsening of nephropathy compared to placebo in type 2 diabetics with a high cardiovascular risk [4]. The trial also revealed a reduction in progression to macroalbuminuria, doubling of the serum creatinine in patients with an estimated glomerular filtration rate (eGFR) less than 45 mL/min/1.73 m^2^, and the requirement of renal-replacement therapy. An initial short-term decrease in the eGFR was noted in diabetic patients on SGLT2 inhibitors. However, this decrease was corrected upon long-term administration of the drug, and thereafter, the eGFR remained stable, while it continued to steadily decline in the placebo group.

Although the CANVAS (Canagliflozin Cardiovascular Assessment Study) trial's primary outcome focused on cardiovascular disease due to its prespecified hypothesis, results showed possible benefits with respect to the progression of albuminuria [5]. Progression of albuminuria, according to the study, was defined as an increase of more than 30% in preexisting albuminuria or a change either from a state of normoalbuminuria to microalbuminuria, from normoalbuminuria to macroalbuminuria, or from microalbuminuria to macroalbuminuria. This trial showed that individuals with type 2 diabetes with a high cardiovascular risk experienced a reduction of 40% in the eGFR as well as the need for renal-replacement therapy (dialysis or transplantation) or death from renal causes (defined as death with a proximate renal cause) after being treated with empagliflozin.

Likewise, the DECLARE (Dapagliflozin and Cardiovascular Outcomes in Type 2 Diabetes) trial's primary outcome focused on cardiovascular disease, and the renal outcome was only secondary [6]. However, results have shown an improvement in renal composite (more than 40% decrease in the eGFR to less than 60 ml/min/1.73 m^2^ of body-surface area, new end-stage renal disease, or death from renal or cardiovascular causes) in individuals with type 2 diabetes with a high cardiovascular risk treated with dapagliflozin compared to those taking placebo. In the overall population, the incidence of the renal composite outcome was 4.3% in the dapagliflozin group versus 5.6% in the placebo group.

The only trial to date to tackle nephropathy as a primary outcome was the recently published CREDENCE trial (Canagliflozin and Renal Outcomes in Type 2 Diabetes and Nephropathy) [7]. The primary outcome of this trial was a composite of end-stage kidney disease (dialysis for at least 30 days, kidney transplantation, or an eGFR <15 ml per minute per 1.73 m^2^ sustained for at least 30 days), doubling of the serum creatinine level from baseline, or death from renal or cardiovascular disease. Type 2 diabetes patients with albuminuria and chronic kidney disease on canagliflozin showed a 30% reduction in primary composite outcomes of end-stage kidney disease, doubling of the serum creatinine level, or renal/cardiovascular death.

Both the CANVAS and EMPA-REG trials revealed a renoprotective effect exerted by SGLT2 inhibitors on kidney function. A reduction in albuminuria was reported which might aid in delaying the progression to renal-replacement therapy. Similar results were shown in the DECLARE trial where dapagliflozin showed more than 40% decrease in the eGFR, a decrease in progression to end-stage kidney disease, and a decrease in death from a renal outcome. However, multiple factors might have contributed to the amelioration of kidney function including improved glycemic control, improved blood pressure, and a decrease in glomerular pressure and volume overload.

Furthermore, the results obtained in the CREDENCE trial support those previously obtained. However, the diabetic patients on canagliflozin enrolled in this trial showed a very modest difference in the blood glucose level, body weight, and blood pressure compared to those on placebo. This suggests that the mechanism of benefit is likely to be independent of glycemic control. Further trials with renoprotective outcomes set as the main hypothesis are due in order to validate the previous results as well as to dwell further on the rationale behind kidney function improvement. Definitive evidence about the effect of multiple SGLT2 inhibitors is likely to be provided in the near future starting with the ongoing and upcoming clinical trials.

Animal studies on SGLT2 inhibitors and diabetic nephropathy were not mentioned in this paper due to the availability of the above clinical trials.

### 2.2. DPP-4 Inhibitors and Nephropathy

The effect of DPP-4 inhibitors on diabetic nephropathy was evaluated in many studies, but few clinical trials about this topic have been conducted so far.

The SAVOR-TIMI 53 trial (Saxagliptin Assessment of Vascular Outcomes Recorded in Patients with Diabetes Mellitus–Thrombolysis in Myocardial Infarction) evaluated the effect of saxagliptin on renal outcomes in diabetic patients with cardiovascular risk factors [8]. At baseline, diabetic patients had normoalbuminuria (urine albumin/creatinine ratio (UACR) <30 mg/g), microalbuminuria (UACR 30–300 mg/g), or macroalbuminuria (UACR >300 mg/g). Treatment with saxagliptin was associated with a statistically significant improvement and/or less deterioration in UACR categories from baseline to the end of the trial (10.7% in the saxagliptin group versus 8.7% in the placebo group). Analyzing the UACR as a continuous variable also showed reduction in albuminuria with saxagliptin. However, the change in the eGFR and serum creatinine and initiation of dialysis were similar in both the treatment and placebo groups.

TECOS (Trial Evaluating Cardiovascular Outcomes with Sitagliptin) is another clinical trial that evaluated chronic kidney disease (CKD) and cardiovascular outcomes in type 2 diabetes individuals with established cardiovascular disease treated with sitagliptin according to their baseline eGFR [9]. Results revealed the same rate decline in kidney function in both the sitagliptin and placebo groups, with a marginally lower but constant eGFR difference. In this study, sitagliptin did not show any clinically significant impact on CKD outcomes, irrespective of the baseline eGFR.

A third trial, the MARLINA-2DM trial (Modification of Albuminuria in Type 2 Diabetes Subjects with Renal Disease with Linagliptin), aimed to investigate the renal effects of linagliptin on individuals with type 2 diabetes with baseline microalbuminuria or moderate macroalbuminuria [10]. Results showed no statistically significant difference in the UACR, although patients treated with linagliptin had a reduction in albuminuria compared to those treated with placebo. This effect, at best modest, was not associated with any major effects on the eGFR.

Finally, the CARMELINA trial (Cardiovascular Safety and Renal Microvascular Outcome Study with Linagliptin) aimed to study the nephroprotective effects of linagliptin on type 2 diabetes patients with a high cardiovascular and renal risk as a secondary outcome [11]. The secondary kidney composite outcome, as defined by the trial, constitutes first-time occurrence of adjunction-confirmed ESRD, death due to renal failure, or a sustained decrease of at least 40% in the eGFR from baseline. Results showed no significant difference between patients on linagliptin (9.4%) and those on placebo (8.8%) with an absolute incidence rate difference of 0.22. The study also had exploratory kidney and microvascular outcomes which comprised sustained ESRD or death due to renal failure or sustained decrease of 50% or more in the eGFR and progression of albuminuria. The results were similar to those of the secondary outcome with no statistically significant difference between the linagliptin and placebo groups. Progression of albuminuria which was defined as the change from normoalbuminuria to microalbuminuria/macroalbuminuria or change from microalbuminuria to macroalbuminuria occurred less frequently in patients on linagliptin (35.3%) than in those on placebo (38.5%) with an absolute incidence rate difference of 3.18.

Compared to other microvascular complications, nephropathy is considered the most studied in major trials on DPP-4 inhibitors including TECOS, SAVOR-TIMI, MARLINA, and CARMELINA. A major conclusion to be drawn from most of these studies, particularly SAVOR-TIMI and CARMELINA, is the positive effect of DPP-4 inhibitors on progression of albuminuria. However, the effect of this medication class on the UACR is still inconclusive as it was not proven to be statistically significant in both the TECOS and MARLINA studies.

CARMELINA showed no significant difference in occurrence of the composite kidney endpoint with linagliptin versus placebo, even after stratifying patients by their baseline characteristics.

Few other studies evaluating the effect of DPP-4 inhibitors on renal function and microalbuminuria have been conducted with some promising results. However, these studies also had major limitations such as a small number of patients, short-duration follow-up, absence of the parallel control group, and results either approaching or completely lacking statistical significance.

Animal studies on DPP-4 inhibitors and diabetic nephropathy were not mentioned in this paper due to the availability of the above clinical trials.

### 2.3. GLP-1 Agonists and Nephropathy

The LEADER (Liraglutide and Cardiovascular Outcomes in Type 2 Diabetes) trial, which assessed the effects of liraglutide on type 2 diabetes subjects with a high cardiovascular risk, showed significant benefits of liraglutide on renal endpoints [12]. Liraglutide was associated with a statistically significant reduction in nephropathy risk, defined as a composite of the onset of macroalbuminuria, persistent doubling of serum creatinine, an eGFR ≤45 mL/min/1.73 m^2^, for continuous renal-replacement therapy, or death from renal disease [13]. The renoprotective effects of liraglutide were mainly driven by the lower incidence of macroalbuminuria [12, 13].

The ELIXA (Evaluation of Lixisenatide in Acute Coronary Syndrome) trial which assessed the effects of lixisenatide on cardiovascular outcomes in patients with type 2 diabetes who had a recent acute coronary event had only analyzed the change in the UACR as a marker of the renoprotective effect [14]. This trial showed a modest difference in the UACR in favor of lixisenatide over placebo.

The SUSTAIN-6 (Semaglutide and Cardiovascular Outcomes in Patients with Type 2 Diabetes) trial, which evaluated the effects of semaglutide, a once weekly GLP-1 analogue, on type 2 diabetes patients with established cardiovascular disease, showed some benefits on renal endpoints [15]. New or worsening nephropathy defined similar to that in the LEADER trial was significantly lower in the semaglutide group with lower macroalbuminuria compared to that in the LEADER trial.

Joined data results from 9 phase II and III trials of the AWARD (Assessment of Weekly Administration of Dulaglutide in Diabetes) study of once weekly dulaglutide did not show a significant difference neither in serum creatinine nor in the eGFR in type 2 diabetes individuals receiving dulaglutide versus those receiving placebo [16]. However, a minor improvement in albuminuria was observed in the dulaglutide group [16].

Study results of the AWARD-7 trial which compares dulaglutide and lispro to glargine and lispro in type 2 diabetes patients with moderate-to-severe CKD revealed favorable outcomes in the former [17]. The eGFR was significantly higher with both dulaglutide doses than with insulin glargine. Moreover, a decline in the eGFR change was significantly smaller for both dulaglutide doses compared with insulin glargine mainly in patients with macroalbuminuria (insulin glargine, –5.5%; dulaglutide 1.5 mg, –0.5%, versus insulin glargine; dulaglutide 0.75 mg, –0.7). On the contrary, decreases from baseline in the UACR were significant within each group but did not differ significantly between groups (insulin glargine, –13.0%; dulaglutide 1.5 mg, –22.5%, versus insulin glargine; dulaglutide 0.75 mg, –20.1%).

Across preclinical and clinical trials, GLP-1 agonists showed renoprotective benefits due to effects on both glomerular endothelium and mesangial cells via the inhibition of the angiotensin II signaling pathway, inhibition of the receptor for AGE (RAGE) gene expression in mesangial cells, and reversal of the abnormal elevation of the oxidative stress markers [18]. Moving to clinical trials, LEADER and SUSTAIN-6 trials showed significant renoprotective effects of liraglutide and semaglutide mainly by reducing macroalbuminuria. Similarly, the most recently published AWARD-7 trial showed renal protection in CKD patients, and such data propose that dulaglutide may have specific therapeutic benefits that can slow progression of moderate-to-severe chronic kidney disease in type 2 diabetes patients, specifically those with macroalbuminuria. In addition, despite the use of an angiotensin-converting enzyme (ACE) inhibitor and an angiotensin receptor blocker (ARB) in most patients in the AWARD-7 trail, albuminuria decreased mostly in the high-dose dulaglutide group suggesting that the effect could be dose related [17]. Note that the key player leading to improvement in the three above trials is the baseline presence of macroalbuminuria. On the contrary, ELIXA and AWARD II/III trials only showed a mild decrease in albuminuria in patients with normal initial kidney function. The latest trial with yet unpublished results is the REWIND (Researching Cardiovascular Events with a Weekly Incretin in Diabetes) trial further evaluating the effect of dulaglutide on renal protection.

However, none of the trials were powered for analysis of the individual renal outcomes, although it was a prespecified secondary outcome in the LEADER, SUSTAIN, and AWARD-7 trials. All trials had a short duration of follow-up ranging from a minimum of 1 year to a maximum of 5 years. There were confounding factors that may have led to renoprotective effects including glucose control and use of other glucose-lowering agents. Future studies with longer durations assessing nephropathy as a primary outcome will help in asserting the renoprotective effect of GLP-1 agonists.

## 3. Neuropathy

### 3.1. SGLT2 Inhibitors and Neuropathy

Neither EMPA-REG nor CANVAS, DECLARE, and CREDENCE trials assessed the effect of SGLT2 inhibitors on neuropathy in type 2 diabetes patients, and so far, no human clinical trials addressed this association. A study conducted by Takakura et al. [19] investigated the effect of another SGLT2 inhibitor ipragliflozin, not yet approved by the FDA, on the progression of neuropathy in diabetic Torii fatty rats. Motor nerve conduction velocity (MNCV) measured on the right sciatic nerve at 24 weeks of age using the Sharma and Thomas method revealed a dose-dependent improvement in rats treated with ipragliflozin versus the diabetic controls. Thus, more trials are needed in order to establish the neuroprotective effect exerted by SGLT2 and whether this class functions to slow down the neurodegenerative process or completely reverse it.

Further studies have revealed that SGLT2 inhibitors reverse glucose-induced vascular dysfunction by reducing glucotoxicity, oxidative stress, and inflammation and also by restoring insulin signaling. The antioxidant and anti-inflammatory effects exerted by SGLT2 inhibitors are most likely due to their glucose-lowering effects as well as the improved glucose utilization by restored insulin sensitivity and signaling [20].

Empagliflozin has been reported to cause reduction in cellular glucotoxicity. It also prevented oxidative stress, advanced glycation end-product (AGE) signaling, and inflammation via NADPH oxidase (NOX) inhibition and decreased the AGE precursor and methylglyoxal. This resulted in maintaining a normal endothelial function in the streptozotocin- (STZ-) treated animal type 1 diabetic model [21].

Thus, the animal trials performed so far revealed promising outcomes in delaying neuropathy. However, human trials need to be conducted in order to assess the reproducibility of these protective outcomes. Moreover, further clinical as well as animal studies should address the mode by which ipragliflozin improves neuropathy: whether through a direct mechanism or by simply controlling blood glucose levels.

### 3.2. DPP-4 Inhibitors and Neuropathy

Currently, there are no available large randomized clinical trials focusing on the effects of DPP-4 inhibitors on diabetic neuropathy. Some studies on this topic have been conducted, although most of them are animal studies. Jin et al. [22] conducted a study to evaluate the effect of vildagliptin on peripheral nerve degeneration in streptozotocin- (STZ-) induced male Sprague Dawley diabetic rats, which was assessed by measuring intraepidermal nerve fiber density changes. Results showed a significant reduction (% change) in the decrease of intraepidermal nerve fiber density in the DPP-4 inhibitor-treated group (normal (10.1%), DM (25.8%), DM with 0.3 mg/kg DPP-IV inhibitor (13.3%), and DM with 10 mg/kg DPP-IV inhibitor (7.9%)).

Another study conducted by Bianchi et al. [23] investigated the protective effects of vildagliptin analogue PKF275-055 on diabetic neuropathy in STZ-induced diabetic rats. They reported that treatment with PKF275-055 restored mechanical sensitivity thresholds by 50% and progressively improved changes in the thermal responsiveness in therapeutic experiments [23, 24].

Tsuboi et al. [25] conducted a study on Goto-Kakizaki (GK) rats with diabetic neuropathy. Vildagliptin was shown to improve both nerve conduction velocity and nerve fiber atrophy, in addition to decreasing intraepidermal nerve fiber density.

In another study performed by Davidson et al. [26], alogliptin was shown to improve nerve conduction velocity in STZ-induced male Sprague Dawley diabetic rats by improving vascular relaxation in epineurial arterioles.

A retrospective cohort study using a large sample from the German electronic medical record database was conducted by Kolaczynski et al. [27] to compare the effect of vildagliptin versus sulfonylurea (SU) on diabetic neuropathy. Treatment with vildagliptin was associated with a significant lower incidence of neuropathy when compared with the SU-treated group.

Another small clinical trial by Da Silva et al. [28] included patients with uncontrolled diabetes on metformin and glyburide who were randomized to receive either sitagliptin or bedtime NPH insulin. It was shown that there was no significant change in sensory and motor nerve conduction parameters in both the two treatment groups after 1 year of follow-up.

Animal studies showed promising beneficial effects of DPP-4 inhibitors on diabetic neuropathy. These studies mainly used vildagliptin, with one study using alogliptin. However, human clinical studies are still scarce in this field. Moreover, the absence of large randomized clinical studies tackling the effects of DPP-4 inhibitors on diabetic neuropathy in the long term makes drawing conclusions about the benefits of DPP-4 inhibitors on neuropathy too preliminary at this point.

### 3.3. GLP-1 Agonists and Neuropathy

Major trials mentioned previously did not assess neuropathy as an outcome. Only one study targeting neuropathy and GLP-1 analogues was conducted in humans. This study included type 2 diabetes patients with mild to moderate diabetic peripheral neuropathy (DPN) who were randomized to receive either exenatide twice daily or glargine [29]. Exenatide did not reduce the prevalence of established DPN, did not affect electrophysiology or measures of small fiber neuropathy, and had no effect on symptoms or signs of DPN. On the contrary, Exendin-4 in preclinical trials showed beneficial effects on diabetic polyneuropathy and peripheral nerve degeneration [30]. In addition, exenatide and liraglutide in animal models with diabetes provided some neuroprotective effects [30].

The effect of GLP-1 agonists on neuropathy is not well studied. Preclinical trials showed benefits of GLP-1 agonists on DPN. The published human study is limited by the small sample size and the short study duration [26]. Thus, the animal studies showing GLP-1 benefits on neuropathy are not be extrapolated to humans. Hence, this complication must be assessed further in human studies through randomized trials with longer follow-up durations.

## 4. Retinopathy

### 4.1. SGLT2 Inhibitors and Retinopathy

Trials so far did not address the effect of SGLT2 inhibitors on diabetic retinopathy. Similar to mesangial kidney cells, retinal pericytes employ SGLT2 receptors for glucose uptake [31]. Studies have shown that, during early phases of diabetic retinopathy (DR), pericytes begin to swell and are eventually lost, resulting in microaneurysm formation, bleeding, and proliferative changes in the retina [32]. A case report by Yoshizumi et al. [33] reported an improvement of visual acuity as well as diabetic macular edema measured by optic coherence tomography in a 63-year-old female with a 7-year history of diabetic retinopathy.

Again the study conducted by Takakura et al. [19] investigated the effect of the SGLT2 inhibitor ipragliflozin on the progression of retinopathy in diabetic Torii fatty rats. Morphological examination of cataract formation was conducted on weekly basis via a slit lamp to measure lens opacity. Wave patterns' peak latency was also measured in these rats via electroretinograms at 18 weeks of age. Diabetic rats not on ipragliflozin showed prolonged peak latencies compared to nondiabetic rats. This prolongation has been reduced dose-dependently in rats on ipragliflozin. Histopathology also revealed that rats on ipragliflozin showed mild lens fiber degeneration and a lower incidence of epithelial hypertrophy/proliferation compared to nontreated diabetics.

To date, limited clinical as well as animal data are available regarding the effect of SGLT2 inhibitors on diabetic retinopathy. Thus, more trials are needed to understand the basis of the limited results at hand and whether the reduction in prolonged peak latency, epithelial hypertrophy, and lens fiber degeneration is a direct effect of SGLT2 inhibitors or is just secondary to glycemic control.

### 4.2. DPP-4 Inhibitors and Retinopathy

Similar to neuropathy, there are no available large randomized clinical trials focusing on the effect of DPP-4 inhibitors on diabetic retinopathy. Available studies are mainly experimental ones conducted on animals.

Gonçalves et al. [34] conducted a study using sitagliptin on STZ-induced diabetic rats (type 1 and 2 diabetes) to test its effect on retinopathy. The breakdown of the blood-retinal barrier (BRB) caused by diabetes was assessed by Evans blue dye. Results showed that treatment with sitagliptin significantly prevented BRB breakdown in diabetic rats as well as decreased the retinal inflammatory state and neuronal apoptosis by a mechanism independent of glycemic control.

Another experimental study was conducted by Maeda el al. [35] using vildagliptin in obese rats with type 2 diabetes. Results showed that treatment with vildagliptin inhibited the overexpression of the genes (vascular endothelial growth factor, intercellular adhesion molecule-1, plasminogen activator inhibitor-1, and pigment epithelium-derived factor) caused by diabetes.

Thus, vildagliptin showed a protective role against diabetic retinopathy by inhibiting inflammatory and thrombogenic reactions in the retinas of these rats.

Dietrich et al. [36] performed a study on STZ-diabetic Wistar rats to test the effect of linagliptin on the retinal neurovascular unit. Rats on linagliptin showed a preventive effect on the loss of pericytes and retinal ganglion cells. The study also revealed a 70% reduction in the increase in acellular capillaries caused by diabetes as well as a 73% reduction of the rise in Iba-1-positive microglia. Thus, the data suggest that linagliptin has a protective effect on the microvasculature of the diabetic retina, most likely due to a combination of neuroprotective and antioxidative effects on the neurovascular unit.

A clinical study conducted by Ott et al. [37] aimed to evaluate the effect of saxagliptin on early retinal microvascular changes by measuring retinal perfusion and pulse wave pressure. Retinal capillary flow (RCF) and central systolic blood pressure were both reduced significantly after treatment with saxagliptin, suggesting a potential effect on improving central hemodynamics and protecting renal microvessels.

Another clinical study was conducted by Chung et al. [38] retrospectively reviewing the medical records of patients with type 2 diabetes and diabetic retinopathy. This study aimed to investigate the effects of DPP-4 inhibitors on the progression of diabetic retinopathy in patients with type 2 diabetes based on the diabetic retinopathy severity scale. Treatment with DPP-4 inhibitors significantly reduced the progression of diabetic retinopathy in patients after propensity score matching when compared to treatment with other oral diabetes medications, independent of glycemic control.

Again, Kolaczynski et al. [27] in their retrospective cohort study using a large sample from the German electronic medical record database also compared the effect of vildagliptin versus sulfonylurea on diabetic retinopathy. Treatment with vildagliptin was associated with a significant lower incidence of retinopathy when compared with the sulfonylurea-treated group in this clinical setting.

Opposing study results were reported by Lee et al. [39] who used various *in vivo* and *in vitro* diabetic retinopathy models. They demonstrated that DPP-4 inhibitors lead to disruption of endothelial cell-to-cell junctions causing increased retinal vascular permeability and leakage. These results, albeit preclinical, raised concerns of the safety of DPP-4 inhibitors with diabetic retinopathy in type 2 diabetes patients.

To date, limited clinical data are available regarding the effect of DPP-4 inhibitors on diabetic retinopathy. However, available data have shown that the use of this class in diabetic patients enhances vascular homeostasis and possibly normalizes early diabetic retinopathy-related hemodynamic changes [18, 39].

A single, opposing preclinical study result would not outweigh the multiple benefits that we have observed from the aforementioned studies to date, but the multiple limitations of these studies do warrant a careful interpretation [18].

### 4.3. GLP-1 Agonists and Retinopathy

The LEADER trial [12] showed that the incidence of retinopathy, defined as the need for retinal photocoagulation or treatment with intravitreal agents, vitreous hemorrhage, or the onset of diabetes-related blindness, was nonsignificantly higher in the liraglutide group than in the placebo group.

The SUSTAIN-6 trial [15] showed a drawback on retinopathy in patients on semaglutide. The semaglutide group had significantly higher retinopathy complications (vitreous hemorrhage, onset of diabetes-related blindness, and the need for treatment with an intravitreal agent or retinal photocoagulation) compared to the placebo group. However, the majority of complications occurred in patients who already had baseline retinopathy [16].

Discrepancy was observed between the results of preclinical and clinical trials. Based on several preclinical trials, GLP-1 and its agonists have a protective effect against diabetic retinopathy through their antiapoptotic and anti-inflammatory mechanisms by reversing and inhibiting early changes, such as neurodegeneration and BRB permeability [18].

On the contrary, the LEADER trial [12] had shown a nonsignificant increase in retinopathy events, while the SUSTAIN-6 trial [15] showed a significant increase in retinopathy incidence. Several factors to explain the discrepancies were listed by Simó and Hernández [40] including the short duration of the trials, absence of grading of diabetic retinopathy, rapid lowering of HbA1c, and a possible direct effect of semaglutide on the retina.

A post hoc analysis of SUSTAIN trails showed no inequity in diabetic retinopathy adverse events across SUSTAIN 1 to 5. The majority of the effect with semaglutide versus placebo in SUSTAIN-6 may be related to the magnitude and speediness of HbA1c reduction early in treatment in patients who had preexisting DR and poor glycemic control at baseline and who were treated with insulin [41]. A retrospective study evaluating the effect of exenatide on diabetic retinopathy showed that exenatide was associated with transient worsening of diabetic retinopathy despite improvement in glycemic control. However, retinopathy improved with continued treatment in the majority of cases [42, 43].

A recent review of the Food and Drug Administration Adverse Event Reporting System (FAERS) hypothesized that semaglutide and retinopathy progression could be due to rapid improvement in blood control [44].

Therefore, abrupt improvement in glycemia may be a trigger for worsening retinopathy in diabetic patients who are poorly controlled. Further trials to evaluate the effects of GLP-1 analogues on retinopathy should be performed, and new guidelines to monitor retinopathy progression in patients on GLP-1 analogues who have baseline diabetic retinopathy and poorly controlled diabetes should be initiated.

## 5. Conclusion

Both SGLT2 inhibitors and GLP-1 agonists appear to have promising nephroprotective outcomes at this stage, with less promising outcomes seen in DPP-4 inhibitors (Table 1). However, more studies are due in order to understand the rationale behind these outcomes and the benefits of these diabetes medications. On the contrary, the neuroprotective outcome has still not been assessed in human clinical trials as neither a primary nor a secondary outcome in the three classes of drugs mentioned in this study. So far, the results at hand are based on a few studies mainly conducted on rats (Table 2). Finally, the retinoprotective effect exerted by both SGLT2 inhibitors and DPP-4 inhibitors was only tested on mice with no human clinical trial conducted yet, while that of GLP-1 agonists was assessed in few trials. However, the results of both GLP-1 agonists and DPP-4 inhibitors showed discrepancies in the different conducted trials (Table 3). All in all, a clearer picture of the microvascular outcomes will manifest over time with the release of the multiple upcoming clinical trials.

## Figures and Tables

**Table 1:** Overview of the renal protective studies.

Authors	Study	Treatment	Size	Duration	Population	Outcome
Wanner and Marx [1]	EMPA-REG OUTCOME	Empagliflozin	7020	3.1 years	DM II High CV risk	Decreased nephropathy
Neal et al. [5]	CANVAS	Canagliflozin	10142	3.6 years	DM II High CV risk	Decreased albuminuria
Marso et al. [12]	LEADER	Liraglutide	9340	3.8 years	DM II High CV risk	Decreased nephropathy and macroalbuminuria
Pfeffer et al. [14]	ELIXA	Lixisenatide	6068	2.1 years	DM II Recent ACS	Decreased UACR
Marso et al. [15]	SUSTAIN-6	Semaglutide	3297	2 yrs	DM II High CV risk	Decreased nephropathy and macroalbuminuria
Tuttle et al. [16]	AWARD II/III	Dulaglutide	6005	0.5 years	DM II	No eGFR change Decreased UACR
Tuttle et al. [17]	AWARD-7	Dulaglutide	577	1 year	DM II Moderate-to-severe CKD	Less eGFR decline in dulaglutide versus glargine Decreased UACR in each group, not significant when compared to glargine
Wiviott et al. [6]	DECLARE	Dapagliflozin	17160	4.2 years	DM II +/– High CV risk	Lower eGFR, ESRD, and death from renal cause
Perkovic et al. [7]	CREDENCE	Canagliflozin	4401	2.62 years	DM II CKD On ACEI	Lower eGFR, ESRD, Cr doubling, and death from renal cause

CV: cardiovascular; ACS: acute coronary syndrome; CKD: chronic kidney disease; ACEI: angiotensin-converting enzyme inhibitor; DM II: diabetes mellitus type 2; eGFR: estimated glomerular filtration rate; ESRD: end-stage renal disease; UACR: urine albumin-to-creatinine ratio; Cr: creatinine.

**Table 2:** Overview of the neurological protective studies.

Authors	Treatment	Size	Duration	Population		Outcome
				Clinical	Animal	
Takakura et al. [19]	Ipragliflozin	44	12 weeks		SDT fatty and SD rats	Reduced prolonged peak latency Improved MNCV
Tsuboi et al. [25]	Vildagliptin	30	18 weeks		Goto-Kakizaki (GK) DM rats	Improved nerve conduction velocity and atrophy
Davidson et al. [26]	Alogliptin	32	12 weeks		STZ-induced DM rats	Improved nerve conduction velocity
Kolaczynski et al. [27]	Vildagliptin	16321	—	DM II		Lower incidence of neuropathy
Da Silva et al. [28]	Sitagliptin	30	1 year	DM II		No benefit on nerve conduction
Jaiswal et al. [29]	Exenatide	42	1.5 years	DM II Mild-to-moderate DPN		No effect on neuropathy

DM II: diabetes mellitus type 2; MNCV: motor neuron conduction velocity; DPN: diabetic peripheral neuropathy; DM: diabetes mellitus; SD: Sprague Dawley; SDT: spontaneously diabetic Torii; STZ: streptozotocin.

**Table 3:** Overview of the ophthalmological protective studies.

Authors/Study	Treatment	Size	Duration	Population		Outcome
				Clinical	Animal	
Takakura et al. [19]	Ipragliflozin	44	12 weeks		SDT fatty and SD rats	Reduced prolonged peak latency
Gonçalves et al. [34]	Sitagliptin	30	4 weeks		STZ-induced diabetic rats	Decreased retinal inflammatory state and neuronal apoptosis Inhibited the BRB breakdown
Maeda et al. [35]	Vildagliptin	26	10 weeks		Obese rats with DM II	Inhibited inflammatory and thrombogenic reactions in the retinas
Dietrich et al. [36]	Linagliptin	44	24 weeks		STZ-induced diabetic rats	Protective effect on the retinal microvasculature
Ott et al. [37]	Saxagliptin	50	6 weeks	DM II		Reduced retinal capillary flow
Chung et al. [38]	DPP-4	82		DM II		Slowed progression of retinopathy
Lee et al. [39]	Sitagliptin	—		DM II High CV risk		Increased retinal vascular permeability and leakage
Marso et al. [12]/LEADER	Liraglutide	9340	3.8 years	DM II High CV risk		Increased retinopathy
Marso et al. [15]/SUSTAIN-6	Semaglutide	3297	2 years	DM II High CV risk		Increased retinopathy
Varadhan et al. [42, 43]	Exenatide	165		DM II		Increased retinopathy and then improved

DM II: diabetes mellitus type 2; DM: diabetes mellitus; SD: Sprague Dawley; SDT: spontaneously diabetic Torii; STZ: streptozotocin; CV: cardiovascular.

## References

[1] Wanner C., Marx N. (2018). SGLT2 inhibitors: the future for treatment of type 2 diabetes mellitus and other chronic diseases. *Diabetologia*.

[2] Mulvihill E. E., Drucker D. J. (2014). Pharmacology, Physiology, and mechanisms of action of dipeptidyl peptidase-4 inhibitors. *Endocrine Reviews*.

[3] Drucker D. J. (2006). The biology of incretin hormones. *Cell Metabolism*.

[4] Zinman B., Wanner C., Lachin J. M. (2015). Empagliflozin, cardiovascular outcomes, and mortality in type 2 diabetes. *New England Journal of Medicine*.

[5] Neal B., Perkovic V., Mahaffey K. W. (2017). Canagliflozin and cardiovascular and renal events in type 2 diabetes. *New England Journal of Medicine*.

[6] Wiviott S. D., Raz I., Bonaca M. P. (2019). Dapagliflozin and cardiovascular outcomes in type 2 diabetes. *New England Journal of Medicine*.

[7] Perkovic V., Jardine M. J., Neal B. (2019). Canagliflozin and renal outcomes in type 2 diabetes and nephropathy. *New England Journal of Medicine*.

[8] Mosenzon O., Leibowitz G., Bhatt D. L. (2017). Effect of saxagliptin on renal outcomes in the SAVOR-TIMI 53 trial. *Diabetes Care*.

[9] Cornel J. H., Bakris G. L., Stevens S. R. (2016). Effect of sitagliptin on kidney function and respective cardiovascular outcomes in type 2 diabetes: outcomes from TECOS. *Diabetes Care*.

[10] Groop P.-H., Cooper M. E., Perkovic V. (2017). Linagliptin and its effects on hyperglycaemia and albuminuria in patients with type 2 diabetes and renal dysfunction: the randomized MARLINA-T2D trial. *Diabetes, Obesity and Metabolism*.

[11] Rosenstock J., Perkovic V., Johansen O. E. (2019). Effect of linagliptin vs placebo on major cardiovascular events in adults with type 2 diabetes and high cardiovascular and renal risk. *JAMA*.

[12] Marso S., Daniels G. H., Brown-Frandsen K. (2016). Liraglutide and cardiovascular outcomes in type 2 diabetes. *New England Journal of Medicine*.

[13] Mann J. F. E., Ørsted D. D., Brown-Frandsen K. (2017). Liraglutide and renal outcomes in type 2 diabetes. *New England Journal of Medicine*.

[14] Pfeffer M. A., Claggett B., Diaz R. (2015). Lixisenatide in patients with type 2 diabetes and acute coronary syndrome. *New England Journal of Medicine*.

[15] Marso S. P., Bain S. C., Consoli A. (2016). Semaglutide and cardiovascular outcomes in patients with type 2 diabetes. *New England Journal of Medicine*.

[16] Tuttle K. R., McKinney T. D., Davidson J. A., Anglin G., Harper K. D., Botros F. T. (2017). Effects of once-weekly dulaglutide on kidney function in patients with type 2 diabetes in phase II and III clinical trials. *Diabetes, Obesity and Metabolism*.

[17] Tuttle K. R., Lakshmanan M. C., Rayner B. (2018). Dulaglutide versus insulin glargine in patients with type 2 diabetes and moderate-to-severe chronic kidney disease (AWARD-7): a multicentre, open-label, randomised trial. *The Lancet Diabetes & Endocrinology*.

[18] Kang Y. M., Jung C. H. (2017). Effects of incretin-based therapies on diabetic microvascular complications. *Endocrinology and Metabolism*.

[19] Takakura S., Toyoshi T., Hayashizaki Y., Takasu T. (2016). Effect of ipragliflozin, an SGLT2 inhibitor, on progression of diabetic microvascular complications in spontaneously diabetic Torii fatty rats. *Life Sciences*.

[20] Cersosimo E., DeFronzo R. A. (2006). Insulin resistance and endothelial dysfunction: the road map to cardiovascular diseases. *Diabetes/Metabolism Research and Reviews*.

[21] Oelze M. (2014 Nov 17). The sodium-glucose co-transporter 2 inhibitor empagliflozin improves diabetes-induced vascular dysfunction in the streptozotocin diabetes rat model by interfering with oxidative stress and glucotoxicity. *PLoS One*.

[22] Jin H. Y., Liu W. J., Park J. H., Baek H. S., Park T. S. (2010). Effect of dipeptidyl peptidase-IV (DPP-IV) inhibitor (Vildagliptin)on peripheral nerves in streptozotocin-induced diabetic rats. *Archives of Medical Research*.

[23] Bianchi R., Cervellini I., Porretta-Serapiglia C. (2012). Beneficial effects of PKF275-055, a novel, selective, orally bioavailable, long-acting dipeptidyl peptidase IV inhibitor in streptozotocin-induced diabetic peripheral neuropathy. *Journal of Pharmacology and Experimental Therapeutics*.

[24] Kawanami D., Matoba K., Sango K., Utsunomiya K. (2016). Incretin-based therapies for diabetic complications: basic mechanisms and clinical evidence. *International Journal of Molecular Sciences*.

[25] Tsuboi K., Mizukami H., Inaba W., Baba M., Yagihashi S. (2016). The dipeptidyl peptidase IV inhibitor vildagliptin suppresses development of neuropathy in diabetic rodents: effects on peripheral sensory nerve function, structure and molecular changes. *Journal of Neurochemistry*.

[26] Davidson E. P., Coppey L. J., Dake B., Yorek M. A. (2011). Treatment of streptozotocin-induced diabetic rats with alogliptin: effect on vascular and neural complications. *Experimental Diabetes Research*.

[27] Kolaczynski W. M., Hankins M., Ong S. H., Richter H., Clemens A., Toussi M. (2016). Microvascular outcomes in patients with type 2 diabetes treated with vildagliptin vs. Sulfonylurea: a retrospective study using German electronic medical records. *Diabetes Therapy*.

[28] Da Silva G., Heise C., Hirata M. (2015). Comparative effects of a dipeptidyl peptidase-4 inhibitor and of NPH insulin on peripheral nerve conduction of patients with type 2 diabetes. *Diabetology & Metabolic Syndrome*.

[29] Jaiswal M., Martin C. L., Brown M. B. (2015). Effects of exenatide on measures of diabetic neuropathy in subjects with type 2 diabetes: results from an 18-month proof-of-concept open-label randomized study. *Journal of Diabetes and Its Complications*.

[30] Seufert J., Gallwitz B. (2014). The extra-pancreatic effects of GLP-1 receptor agonists: a focus on the cardiovascular, gastrointestinal and central nervous systems. *Diabetes, Obesity and Metabolism*.

[31] Mandarino L. J., Finlayson J., Hassell J. R. (1994). High glucose downregulates glucose transport activity in retinal capillary pericytes but not endothelial cells. *Investigative Ophthalmology and Visual Science*.

[32] Armulik A., Abramsson A., Betsholtz C. (2005). Endothelial/pericyte interactions. *Circulation Research*.

[33] Yoshizumi H., Ejima T., Nagao T., Wakisaka M. (2018). Recovery from diabetic macular edema in a diabetic patient after minimal dose of a sodium glucose co-transporter 2 inhibitor. *American Journal of Case Reports*.

[34] Gonçalves A., Marques C., Leal E. (2014). Dipeptidyl peptidase-IV inhibition prevents blood–retinal barrier breakdown, inflammation and neuronal cell death in the retina of type 1 diabetic rats. *Biochimica et Biophysica Acta*.

[35] Maeda S., Yamagishi S.-I., Matsui T. (2013). Beneficial effects of vildagliptin on retinal injury in obese type 2 diabetic rats. *Ophthalmic Research*.

[36] Dietrich N., Kolibabka M., Busch S. (2016). The DPP4 inhibitor linagliptin protects from experimental diabetic retinopathy. *PLoS One*.

[37] Ott C., Raff U., Schmidt S. (2014). Effects of saxagliptin on early microvascular changes in patients with type 2 diabetes. *Cardiovascular Diabetology*.

[38] Chung Y.-R., Park S. W., Kim J. W., Kim J. H., Lee K. (2016). Protective effects of dipeptidyl peptidase-4 inhibitors on progression of diabetic retinopathy in patients with type 2 diabetes. *Retina*.

[39] Lee C. S., Kim Y. G., Cho H.-J. (2016). Dipeptidyl peptidase-4 inhibitor increases vascular leakage in retina through VE-cadherin phosphorylation. *Scientific Reports*.

[40] Simó R., Hernández C. (2017). GLP-1R as a target for the treatment of diabetic retinopathy: friend or foe?. *Diabetes*.

[41] Vilsbøll T., Bain S. C., Leiter L. A. (2018). Semaglutide, reduction in glycated haemoglobin and the risk of diabetic retinopathy. *Diabetes, Obesity and Metabolism*.

[42] Varadhan L., Humphreys T., Hariman C., Walker A. B., Varughese G. I. (2011). GLP-1 agonist treatment: implications for diabetic retinopathy screening. *Diabetes Research and Clinical Practice*.

[43] Varadhan L., Humphreys T., Walker A. B., Varughese G. I. (2014). The impact of improved glycaemic control with GLP-1 receptor agonist therapy on diabetic retinopathy. *Diabetes Research and Clinical Practice*.

[44] Fadini G. P., Sarangdhar M., Avogaro A. (2018). Glucagon-like peptide-1 receptor agonists are not associated with retinal adverse events in the FDA adverse event reporting system. *BMJ Open Diabetes Research & Care*.

